# Actin-dependent mechanisms in AMPA receptor trafficking

**DOI:** 10.3389/fncel.2014.00381

**Published:** 2014-11-12

**Authors:** Jonathan G. Hanley

**Affiliations:** School of Biochemistry, University of BristolBristol, UK

**Keywords:** synaptic plasticity (LTP/LTD), endocytosis, exocytosis, glutamate receptor, dendritic spine

## Abstract

The precise regulation of AMPA receptor (AMPAR) number and subtype at the synapse is crucial for the regulation of excitatory neurotransmission, synaptic plasticity and the consequent formation of appropriate neural circuits for learning and memory. AMPAR trafficking involves the dynamic processes of exocytosis, endocytosis and endosomal recycling, all of which involve the actin cytoskeleton. The actin cytoskeleton is highly dynamic and highly regulated by an abundance of actin-binding proteins and upstream signaling pathways that modulate actin polymerization and depolymerization. Actin dynamics generate forces that manipulate membranes in the process of vesicle biogenesis, and also for propelling vesicles through the cytoplasm to reach their destination. In addition, trafficking mechanisms exploit more stable aspects of the actin cytoskeleton by using actin-based motor proteins to traffic vesicular cargo along actin filaments. Numerous studies have shown that actin dynamics are critical for AMPAR localization and function. The identification of actin-binding proteins that physically interact with AMPAR subunits, and research into their mode of action is starting to shed light on the mechanisms involved. Such proteins either regulate actin dynamics to modulate mechanical forces exerted on AMPAR-containing membranes, or associate with actin filaments to target or transport AMPAR-containing vesicles to specific subcellular regions. In addition, actin-regulatory proteins that do not physically interact with AMPARs may influence AMPAR trafficking by regulating the local actin environment in the dendritic spine.

## Introduction

AMPA receptors (AMPARs) mediate the majority of fast synaptic excitation in the brain. Therefore, the precise regulation of AMPAR number and subtype at the synapse is crucial to excitatory neurotransmission, synaptic plasticity and the consequent formation of appropriate neural circuits during learning and memory. AMPAR trafficking involves the dynamic processes of exocytosis, endocytosis and endosomal recycling. In addition, receptors may be restricted in their movement to maintain their localization at the postsynaptic membrane, or at intracellular compartments. The molecular mechanisms that underlie AMPAR trafficking under basal conditions and during certain forms of synaptic plasticity are the topics of very active research and some excellent review articles (Shepherd and Huganir, 2007; Henley et al., 2011; Van Der Sluijs and Hoogenraad, 2011; Anggono and Huganir, 2012).

The actin cytoskeleton is highly dynamic and highly regulated. It is composed of monomeric globular (G)-actin, which polymerizes to form actin filaments (F-actin), and an abundance of actin-binding proteins and upstream signaling pathways regulate actin polymerization and depolymerization. Depending on the relative activity of these multiple regulatory mechanisms, adenosine triphosphate (ATP)-bound G-actin monomers are polymerized at the plus (or “barbed”) end of an actin filament, and adenosine diphosphate (ADP)-bound monomers depolymerize from the minus (or “pointed”) end (Lee and Dominguez, 2010). This process is known as actin “treadmilling” and can generate forces to bring about movement in the cell. The actin cytoskeleton plays critical roles in cell morphology and cell motility, ie defining the shape and movement of the entire cell (Pollard and Cooper, 2009). Actin dynamics are also used by the cell to generate forces that manipulate membranes in the process of vesicle biogenesis, and also for propelling vesicles and larger endosomal compartments through the cytoplasm to reach their destination. For example, the role of the dynamic actin cytoskeleton in endocytosis has been intensively studied in non-neuronal cells, and a highly complex mechanism involving numerous actin-regulatory molecules involved in this process is emerging (Galletta and Cooper, 2009; Mooren et al., 2012). SNARE-mediated membrane fusion events are required for the final insertion of receptors into the plasma membrane in the process of exocytosis. This is also an active process that has been shown to require actin dynamics (Porat-Shliom et al., 2013). In addition, trafficking mechanisms exploit more stable aspects of actin by using actin-based motor proteins to traffic vesicular cargo along actin filaments (Kneussel and Wagner, 2013).

**Figure 1:**
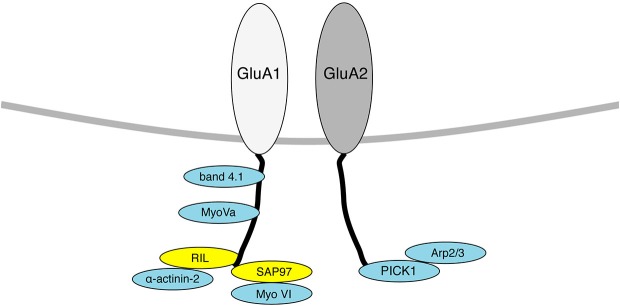
**Diagram indicating AMPAR interacting proteins that also associate with the actin cytoskeleton**. Proteins that bind directly to F-actin are shown in blue, proteins that act as a scaffold to bring actin binding proteins to GluA1/2 are shown in yellow. Only proteins that have a known physical association with AMPAR subunits are shown here.

Following a brief review of early work demonstrating the importance of the actin cytoskeleton in AMPAR trafficking, this paper will focus on proteins that either bind to or regulate the actin cytoskeleton to influence AMPAR trafficking. Many of these proteins physically associate with AMPAR subunits (Figure 1). In some cases, the evidence indicates that a specific protein plays a critical role in regulating AMPAR trafficking or localization, but the precise trafficking event that is affected, or the mechanistic details of the interaction with the actin cytoskeleton are unclear.

Dendritic spines are highly enriched in dynamic actin filaments, reflecting the highly plastic nature of this subcellular compartment. The spine actin cytoskeleton has an important structural role, since actin polymerization is associated with spine enlargement, and depolymerization with spine shrinkage. (Bosch and Hayashi, 2012; Fortin et al., 2012). The actin-dependent mechanisms that underlie spine structural plasticity are outside the scope of this paper, although some actin-based pathways play a role in regulating both AMPAR trafficking and structural plasticity (Fukazawa et al., 2003; Gu et al., 2010; Rocca et al., 2013; Bosch et al., 2014). The spine is a highly active trafficking compartment, so a high concentration of dynamic F-actin in the spine is well-placed to regulate various aspects of receptor trafficking, especially that of AMPARs. Early indications that the actin cytoskeleton is involved in AMPAR localization at the synapse came from studies using actin depolymerizing drugs such as latrunculin. Cultured neurons exposed to latrunculin showed reduced clustering of GluA1-containing AMPARs in dendritic spines (Allison et al., 1998), and reduced surface expression at synapses (Kim and Lisman, 1999; Zhou et al., 2001). Moreover, the F-actin stabilizing drug Jasplakinolide blocked ligand-stimulated AMPAR internalization (Zhou et al., 2001). Taken together, these studies showed that F-actin is involved in maintaining AMPARs at synapses, and also that actin depolymerization is required for the removal of AMPARs from the synaptic plasma membrane. Further studies showed that long term potentiation (LTP) was blocked in the presence of either latrunculin or phalloidin, which is another F-actin stabilizing agent, indicating that the dynamic actin cytoskeleton is required for the potentiation of AMPAR function (Kim and Lisman, 1999). A later report from the same authors suggested that although a pool of AMPARs is dynamically regulated by the actin cytoskeleton, a distinct pool remains stable in the presence of actin-disrupting agents, and therefore may not be regulated by actin (Kim and Lisman, 2001).

## Actin-associated proteins that regulate AMPAR localization or trafficking

### Protein 4.1

One initial interpretation of these data was that AMPARs are somehow anchored to actin filaments at the synapse to cluster them at the postsynaptic density (PSD), restricting their lateral mobility and their endocytosis from the plasma membrane. This would implicate either a direct interaction between AMPAR subunits and actin filaments, or the involvement of linker proteins that would mediate such an association. Around the same time as these studies, a number of labs were characterizing novel AMPAR protein interactions that had been discovered by yeast-2-hybrid screens, and the discovery that AMPAR subunit GluA1 interacts directly with the F-actin-associated proteins 4.1N and 4.1G provided a molecular mechanism for this model (Shen et al., 2000). 4.1 proteins were originally identified in erythrocytes and are critical for the organization of the spectrin–actin cytoskeleton and for the association of the cytoskeleton with membranes via interaction with membrane proteins (Baines et al., 2014). A role for 4.1G/N in actin-dependent AMPAR localization was demonstrated in experiments with GluA1 lacking the 4.1G/N binding site, which showed reduced surface expression in biotinylation assays, and this mutation occluded the effects of latrunculin treatment (Shen et al., 2000). Although these experiments were carried out in heterologous cells and not in neurons, 4.1G/N appeared to perform the role of stabilizing surface AMPARs by providing a link with actin filaments. In contrast to the static anchoring function proposed by Shen et al., a dynamic role for 4.1 proteins was suggested by a more recent study using live imaging of super-ecliptic pHluorin (SEP)-tagged GluA1 in conjunction with TIRF microscopy. SEP is a pH-sensitive variant of GFP that fluoresces at neutral pH (cell surface), and is quenched at low pH (intracellular vesicles/endosomes) (Ashby et al., 2004). This technique enables the anlaysis of GluA1 insertion events in real time, which are visualized as fluorescent puncta that rapidly appear and then dissipate gradually over time as the receptors diffuse away from the site of insertion. Knockdown of 4.1N expression using shRNA caused a reduction in the frequency of insertion events at extrasynaptic sites. While the actin cytoskeleton *per se* was not studied in this paper, the data suggest a role for 4.1N in AMPAR exocytosis rather than in surface stability (Lin et al., 2009). Based on this dynamic model, disrupting 4.1 protein function would still cause reduced AMPAR surface levels because of reduced receptor insertion into the plasma membrane. The precise molecular mechanism that underlies such a dynamic role for 4.1N is unclear. These observations were questioned by a report of a mutant mouse expressing only 22% of wild-type 4.1N levels and lacking 4.1G entirely. While synaptosomal levels of GluA1 were modestly reduced in the double “knockout”, both basal synaptic transmission and LTP in CA1 region of hippocampal slices were unaffected, suggesting that the 4.1-mediated link with the actin cytoskeleton is dispensable for AMPAR localization at the synapse and for regulated trafficking (Wozny et al., 2009).

### RIL/α-Actinin-2

Another AMPAR interacting protein that associates with the actin cytoskeleton is RIL (reversion-induced LIM protein), which binds GluA1 C-terminus and also the F-actin cross-linking protein α-actinin-2. Although this interaction is not well-characterized, it is proposed to play a role in enhancing surface and synaptic expression of AMPARs by regulating endosomal recycling (Schulz et al., 2004). Exogenously expressed RIL colocalizes with transferrin receptors (TfR) in COS cells and enhances the localization of exogenous GluA1 to TfR positive compartments. In neurons, RIL overexpression causes increased enrichment of AMPARs in dendritic spines, and increased mEPSC amplitude. It is unclear whether this occurs via dynamic control of the actin cytoskeleton, or via the targeting of AMPAR-containing endosomes to actin filaments. A possible mechanism might be that RIL mediates an interaction between endosomal GluA1 and actin filaments, and myosin-based transport shuttles the recycling receptors to the plasma membrane (see following section). Indeed, actinin-4 has been shown to function in complex with MyoV to regulate transferrin receptor recycling (Yan et al., 2005). However, RIL has been reported to influence F-actin dynamics in non-neuronal cells (Vallenius et al., 2004), suggesting that alternative mechanisms could be involved.

### Myosin motor proteins

An important aspect of AMPAR trafficking is the transport of AMPAR-containing vesicles or larger endosomal compartments to and from the sites of exo- and endocytosis respectively. Myosins are actin-based motor proteins that hydrolyze ATP to generate mechanical force, which is directed as movement along actin filaments (Soldati and Schliwa, 2006). Various myosin isoforms are involved in AMPAR trafficking, the best characterized being MyoV and MyoVI, which are plus end directed and minus end directed motors, respectively. Plus end directed motors move towards the barbed (plus) end of actin filaments, and hence tend to direct cargo to the cell periphery. In contrast, minus end directed motors move towards the pointed (minus) end of actin filaments, and a major role is in the movement of endocytic vesicles away from the plasma membrane (Hartman et al., 2011). MyoVI is a minus-end directed motor that associates with AMPARs via the scaffold protein SAP97, which in turn binds GluA1 via a PSD-95/discs large/zona occludens (PDZ) interaction (Leonard et al., 1998; Wu et al., 2002). These early studies used biochemical techniques to describe the protein interactions, and it was later shown that this complex also contains the endocytic adaptor protein AP2, and that AMPAR internalization stimulated by insulin or by AMPA is abolished in cultured neurons from MyoVI knockout mice (Osterweil et al., 2005). This suggests a role for MyoVI in AMPAR internalization, probably in the transport of endocytic vesicles from the sites of endocytosis at the plasma membrane to endosomal compartments. A more recent study used a C-terminal fragment of MyoVI as a dominant negative to disrupt endogenous MyoVI-SAP97 interactions. In contradiction to Wu et al. and Osterweil et al., neurons expressing this construct show reduced surface expression of endogenous AMPARs analyzed by immunocytochemistry in cultured hippocampal neurons (Nash et al., 2010). The same treatment causes a complete block of NMDAR-dependent AMPAR insertion at the plasma membrane stimulated by brief (3 × 1 s) transient depolarization.

In contradiction to Osterweil et al., these results suggest a role for MyoVI in trafficking AMPARs towards the plasma membrane, rather than in internalization. A possible explanation is that the C-terminal fragment of MyoVI used by Nash et al. binds to and hence blocks interactions with the N-terminus of SAP97, which includes the L27 domain (Wu et al., 2002). SAP97 is a multi-functional scaffold protein, and multimerization via the L27 domain has been suggested to be required for AMPAR targeting to the synapse (Nakagawa et al., 2004).

Two distinct myosin motor-dependent mechanisms have been suggested for the forward traffic of AMPARs to the synapse in response to LTP induction. MyoVa can bind directly to the GluA1 C-terminus, and is required for LTP in CA1 neurons of organotypic slice cultures, but not for constitutive AMPAR trafficking under basal conditions, which was also assayed electrophysiologically (Correia et al., 2008). In contrast, biochemical experiments showed that MyoVb interacts with the recycling endosome protein complex Rab11-FIP2 in a Ca^2+^ dependent manner (Wang et al., 2008). A direct interaction between MyoVb and AMPAR subunits was not tested in this study. Time-lapse imaging of fluorescently-tagged TfR expressed in cultured hippocampal neurons demonstrated that MyoVb promotes the entry of recycling endosomes into dendritic spines in response to chemical LTP induction. Experiments using SEP-GluA1 to report surface accumulation of exogenous AMPARs showed that this mechanism is required for the surface delivery of AMPARs in response to chemical LTP (Wang et al., 2008). The same authors also used electrophysiological techniques to demonstrate a requirement for MyoVb in LTP in hippocampal CA1 neurons. Another report suggested that MyoVb also affects surface AMPAR expression under basal conditions by expressing a mutant form of MyoVb lacking the region required for interacting with Rab11, and analyzing surface expression of endogenous GluA1 (Lise et al., 2006). Interestingly, MyoVa is also regulated by Ca^2+^ (Wang et al., 2004), although specific Ca^2+^-dependent interactions relevant to AMPAR trafficking have not been revealed. Furthermore, MyoVa dominant negative reduces Rab11 localization to spines, suggesting that it may too have general effects on recycling endosomal entry into spines (Correia et al., 2008). Whether both MyoVa and MyoVb do indeed play critical yet subtly different roles in AMPAR trafficking to synapses, or the different results reflect the use of different experimental approaches (dominant negative MyoVa vs. siRNA for MyoVb) that disrupt the same mechanism is unclear.

### ADF/Cofilin

Once AMPAR-containing vesicles or endosomes reach the postsynaptic membrane, SNARE-mediated membrane fusion events are required for the incorporation of receptors into the plasma membrane. Although specific SNARE proteins and specific plasma membrane domains for AMPAR insertion have been identified (Kennedy et al., 2010; Jurado et al., 2013), little is known about the specific machinery that regulates the actin cytoskeleton during these events. However, a role for actin dynamics in AMPAR insertion into the spine plasma membrane has been demonstrated. Actin Depolymerizing Factor (ADF)/cofilin is a ubiquitous actin-binding protein, which is involved in the reorganization of actin filaments by causing depolymerization of F-actin at the minus end of filaments, and also by severing actin filaments (Sarmiere and Bamburg, 2004). Hence cofilin reduces the proportion of F-actin, but also increases the pool of G-actin available for subsequent polymerization, hence increasing F-actin turnover. Cofilin activity is tightly regulated by phosphorylation at Serine 3; phosphorylation by LIM kinase deactivates the protein, and dephosphorylation of the same site by Slingshot phosphatases activates cofilin (Mizuno, 2013). The expression of cofilin phosphorylation mutants that either constitutively activate or deactivate cofilin demonstrated that activated cofilin is required for the insertion of SEP-GluA1 into the spine plasma membrane following the chemical induction of LTP in live imaging experiments in cultured neurons (Gu et al., 2010). Consistent with a role for cofilin in enhancing AMPAR synaptic expression, Slingshot knockdown by RNAi causes a reduction in AMPAR EPSC frequency and amplitude in both dissociated cultures and organotypic slice cultures, and also blocks synaptic plasticity induced by infusion of active CaMKII (Yuen et al., 2010). A requirement for cofilin in LTP was also demonstrated by the generation of cofilin knockout mice, which showed a complete lack of LTP in CA1 neurons of hippocampal slices (Rust et al., 2010). In this paper, LTP-induced increases in surface-expressed AMPARs were not investigated, but instead cofilin was shown to play a role in AMPAR surface diffusion, which was studied using single-particle tracking of endogenous AMPARs labeled with quantum dot conjugated antibodies. It is well-established that synaptic AMPARs show markedly less diffusion at synaptic compared to extrasynaptic sites (Opazo and Choquet, 2011). While the lateral diffusion of AMPARs at synaptic sites was unaffected, extrasynaptic receptors were significantly less mobile in the absence of cofilin (Rust et al., 2010). Hence, cofilin appears to be involved in AMPAR trafficking to the synapse by regulating exocytosis and also by modulating the surface diffusion of extrasynaptic receptors, which affects the probability of a surface-expressed receptor being incorporated into the synapse (Opazo and Choquet, 2011). Further work will be needed to determine whether these observations reflect a general requirement for increased actin turnover during these dynamic trafficking events in the spine, or whether cofilin physically interacts with AMPARs or associated scaffold proteins to mediate temporally and locally precise changes in actin dynamics.

The role of actin dynamics in AMPAR lateral mobility was studied further by Kerr and Blanpied, who employed high-resolution FRAP (fluorescence recovery after photobleaching) of SEP-GluA1 and also of PSD scaffold proteins to determine their mobility within the PSD, and analyzed the effects of pharmacological manipulations of the actin cytoskeleton. These experiments suggested that while AMPARs show very little free diffusion within the PSD, they are restricted to spatial subdomains that are defined by subsynaptic scaffolds and the actin cytoskeleton. Actin dynamics causes remodeling of the underlying scaffold, which in turn causes continuous spatial readjustments of AMPAR subdomains and hence their positioning within the PSD (Kerr and Blanpied, 2012). The actin-regulatory machinery responsible for regulating the dynamics of scaffold proteins and AMPARs in the PSD were not investigated in this study. Another important finding from this paper was that loss of actin filaments had only a very small effect on AMPAR synaptic localization within the timescale of the experiment. A dramatic loss of F-actin was observed after just 5 min of latrunculin treatment, but only a small (yet still increasing) loss of SEP-tagged AMPAR subunits was recorded at 10 min (Kerr and Blanpied, 2012). This result does not support a role for actin filaments in simply anchoring AMPARs at the synapse, but instead may reflect a role for actin dynamics in regulating receptor trafficking events at extrasynaptic sites.

### PICK1 and the ARP2/3 complex

As well as being involved in the maintenance or increase in AMPAR surface expression at the synapse, the dynamic regulation of the actin cytoskeleton is also involved in reducing the levels of surface-expressed AMPARs. The actin-nucleating Arp2/3 complex is the major catalyst for the formation of branched actin networks that mediate changes in membrane geometry (Campellone and Welch, 2010). Proteins such as N-WASP, WAVE and related proteins bind and activate the Arp2/3 complex, and are highly regulated so that changes in cell morphology or vesicle trafficking occur at appropriate times and subcellular locations (Takenawa and Suetsugu, 2007). PICK1 binds GluA2/3 subunits via its PDZ domain, and plays a critical role in reducing AMPAR surface expression during LTD (Kim et al., 2001; Terashima et al., 2008). PICK1 also binds directly to F-actin via the BAR domain, and to the Arp2/3 complex via a C-terminal portion of the protein including a critical tryptophan residue, W413. These interactions were defined by *in vitro* assays using purified protein components as well as co-immunoprecipitations from native tissue (Rocca et al., 2008). In *in vitro* pyrene-tagged actin polymerization assays, PICK1 inhibits Arp2/3-mediated actin nucleation and polymerization, and both F-actin and Arp2/3 interactions are required for this inhibitory activity (Rocca et al., 2008). PICK1 inhibits the Arp2/3 complex by competing with Arp2/3 activators such as N-WASP for binding to the complex, but also has a direct inhibitory effect that can be observed in the absence of other proteins in the in vitro pyrene assay. In antibody-feeding immunocytochemistry experiments, molecular replacement with a PICK1 W413A mutant blocks AMPAR internalization in response to chemical LTD induction in cultured neurons (Rocca et al., 2008). Furthermore, the same mutant blocks CA1 LTD in hippocampal slices (Nakamura et al., 2011). These results demonstrate that the inhibition of Arp2/3-mediated actin polymerization by PICK1 is required for AMPAR internalization. However, it is unclear whether PICK1 functions mainly at the level of the plasma membrane to promote AMPAR endocytosis, or at the recycling endosome to restrict AMPAR recycling, or both. Both trafficking processes involve BAR domain proteins that are involved in bending or tubulating membranes, and both also involve actin dynamics as regulators of mechanical force to control vesicle formation or tubulation (Galletta and Cooper, 2009; Van Der Sluijs and Hoogenraad, 2011; Mooren et al., 2012; Suetsugu and Gautreau, 2012).

The PICK1-F-actin and the PICK1-Arp2/3 complex interactions are modestly auto-inhibited by an intramolecular interaction between the PDZ domain and the BAR domain, and Arp2/3 inhibition is enhanced by the binding of a GluA2 C-terminal fragment to the PICK1 PDZ domain in *in vitro* actin polymerization assays (Rocca et al., 2008). This suggests a mechanism to ensure that maximal Arp2/3 inhibition is temporally and spatially focused to promote PICK1-mediated AMPAR trafficking. PICK1 is a Ca^2+^ sensor that responds to NMDAR-mediated Ca^2+^ influx to enhance its interaction with GluA2 (Hanley and Henley, 2005), hence actin dynamics in the vicinity of AMPARs are modulated by NMDAR stimulation in response to the induction of synaptic plasticity. A further level of regulation is provided by the small GTPase Arf1, which interacts directly with PICK1 to reduce Arp2/3 binding and consequent inhibition of Arp2/3 activity in *in vitro* assays. (Rocca et al., 2013). Arf1 appears to inhibit PICK1 under basal conditions, since molecular replacement with an Arf1 mutant that does not bind PICK1 causes the loss of surface AMPARs in cultured neurons, which occludes subsequent NMDA-induced internalization. Arf1 binds PICK1 preferentially in its active, guanosine triphosphate (GTP)-bound state, hence a signaling event to increase PICK1-mediated Arp2/3 inhibition to promote trafficking would require a switch from GTP-bound to GDP-bound Arf1. Indeed, a reduction in GTP bound Arf1 in response to NMDAR stimulation in cultured neurons is blocked by siRNA-mediated knockdown of the Arf GAP GIT1 (Rocca et al., 2013), which is a protein previously implicated in AMPAR trafficking (Ko et al., 2003). This model also suggests the involvement of an Arf1 GEF to maintain a basal level of GTP-Arf1 and hence a low basal level of Arp2/3 inhibition by PICK1 at appropriate subcellular locations, presumably close to synapses. Further work will determine the spatial organization of this process and the precise mechanism of NMDAR-stimulated Arf GAP activity by GIT1.

### ARC/ARG3.1

Arc/Arg3.1 associates with the actin cytoskeleton, but does not bind actin directly, and the intermediate protein involved in this interaction is unknown. Arc also interacts with the endocytic proteins endophilin and dynamin (Chowdhury et al., 2006) to promote AMPAR internalization during homeostatic synaptic plasticity in cultured neurons and certain forms of memory (Shepherd et al., 2006; Liu et al., 2012). None of these proteins binds AMPAR directly, and the specific molecular interactions involved in AMPAR trafficking, including a potential role for the association of Arc with actin, are unclear. An interesting observation linking Arc to actin dynamics is that Arc synthesis causes cofilin phosphorylation (Messaoudi et al., 2007). Since phosphorylated cofilin is inactive, this suggests that Arc synthesis has a stabilizing influence on actin dynamics. Cofilin has not been implicated in AMPAR internalization *per se*, so this influence of Arc on actin dynamics may be independent of its role in AMPAR endocytosis.

### CPG2

Another actin-associated protein involved in regulating AMPAR internalization is CPG2 (Candidate Plasticity Gene 2), which binds F-actin directly and colocalizes with clathrin at postsynaptic endocytic zones (Cottrell et al., 2004; Loebrich et al., 2013). CPG2 knockdown causes an increase in surface-expressed AMPARs, an increase in synaptic strength, and an accumulation of clathrin-coated vesicles close to synapses, suggesting that it may play a role in a late phase of endocytosis, such as vesicle movement away from the plasma membrane (Cottrell et al., 2004; Loebrich et al., 2013). Consistent with this hypothesis, F-actin dynamics are thought to play a critical role in the late stages of clathrin-coated pit invagination and subsequent vesicle mobilization (Merrifield, 2004). The CPG2-actin interaction is enhanced by PKA phosphorylation of CPG2, and expression of phospho-null mutant CPG2 reduces basal AMPAR internalization, suggesting that actin binding is required for trafficking. However, the specific function of the actin binding property of CPG2 with respect to AMPAR trafficking is currently unknown.

## Concluding remarks

**Figure 2:**
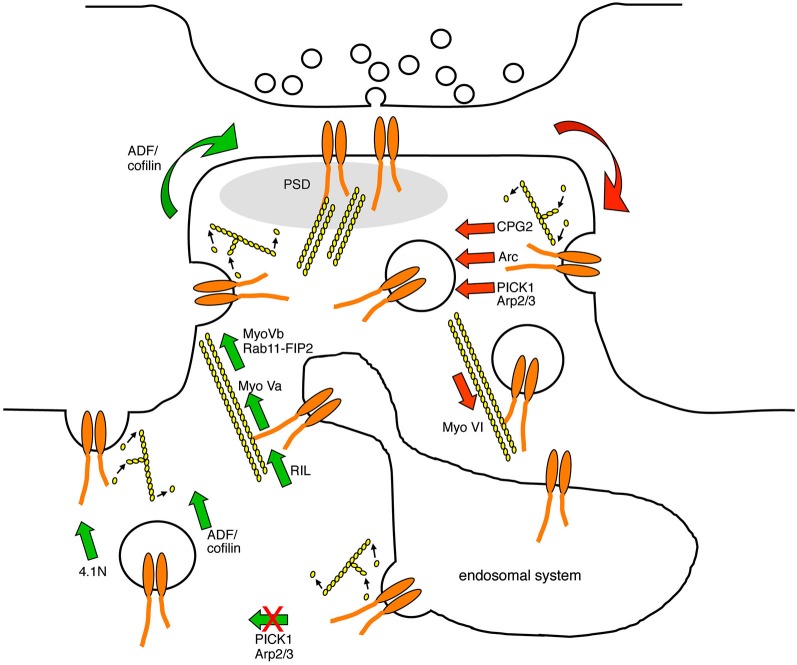
**Diagram indicating the points in the AMPAR trafficking pathway that are known to be regulated by actin-based protein machinery**. AMPARs are endocytosed at endocytic zones adjacent to the PSD in a process that involves the modulation of F-actin turnover, and the actin-biding proteins CPG2, Arc, and the Arp2/3 inhibitor PICK1. AMPAR-containing endocytic vesicles are transported away from the plasma membrane along F-actin tracks by the minus-end directed actin motor protein myosin VI. In the recycling endosome, AMPARs associate with actin filaments via RIL and the plus-end directed motor protein myosin Va, which direct AMPAR traffic towards the plasma membrane. In addition, AMPAR-containing recycling endosomes associate with the plus-end directed motor protein myosin Vb via Rab11-FIP2, which pulls the endosome into the spine to increase the availability of AMPARs for subsequent plasma membrane insertion. PICK1 restricts AMPAR recycling back to the plasma membrane in a process that is likely to involve F-actin turnover. The insertion of AMPARs into the plasma membrane requires the activity of ADF/cofilin and the actin-binding protein 4.1N. Red arrows represent trafficking events that are involved in reducing AMPAR surface expression, and green arrows represent trafficking events that are involved in increasing AMPAR surface expression.

It is clear that the actin cytoskeleton plays a critical role in controlling the dynamic localization of AMPARs, by regulating multiple points in the trafficking pathway (Figure 2). However, the mechanistic details are still far from clear. Fundamental aspects of receptor trafficking have been defined in non-neuronal mammalian cells, which will probably give further clues about the mechanisms at play in neurons to regulate AMPARs. However, the atypical environment of the dendritic spine, which is a small, confined compartment with a very high concentration of dynamic actin filaments, suggests that actin-dependent receptor trafficking mechanisms may involve characteristics that are specific to this environment. Actin-binding proteins or actin-regulatory proteins that associate with AMPAR subunits or with postsynaptic scaffolds have already been shown to be critical regulators of AMPAR trafficking, but a more complete understanding of the spatial and temporal regulation of actin dynamics in relation to AMPARs and to the PSD is necessary. In addition, further work is needed to unravel the upstream regulation of these mechanisms to drive AMPAR trafficking leading to changes in synaptic strength.

## Conflict of interest statement

The author declares that the research was conducted in the absence of any commercial or financial relationships that could be construed as a potential conflict of interest.
